# Editorial: Advancing molecular diagnostic tools for robust surveillance of microbial water quality

**DOI:** 10.3389/fmicb.2024.1452943

**Published:** 2024-07-10

**Authors:** Lisa Paruch, Cristina García-Aljaro

**Affiliations:** ^1^Division of Environment and Natural Resources, Department of Hydrology and Water Environment, Norwegian Institute of Bioeconomy Research, Ås, Norway; ^2^Department of Genetics, Microbiology and Statistics, University of Barcelona, Barcelona, Spain

**Keywords:** microbial water quality, genetic microbial source tracking, microbial risk assessment, waterborne pathogen, aquatic ecosystem

Microbial water quality is of critical importance for human, animal, and environmental health. The presence of microbial pollutants, such as pathogenic bacteria, viruses, protozoa, fungi and associated antimicrobial resistance (AMR) can deteriorate the safety and quality of water to varying extents, including surface water, marine water, ground water, and drinking water, etc. In certain severe cases, waterborne outbreaks occur and can result in significant economic and social losses, which underscores the need for the development and application of rapid, sensitive and reliable survey methods for early detection of microbial pollutants in water to enable a timely response and adopt measures to quickly control and limit the health impact of the pollution.

Rapid and reliable detection of microbial contamination is pivotal for effective management of water quality and for preventing the spread of harmful microbial hazards. Additionally, water pollutants can significantly alter microbial communities in water bodies, disrupting the balance of aquatic ecosystems. Detecting such changes is essential for the prevention of the degradation of aquatic ecosystems, which compromises biodiversity and the ability of nature to maintain essential life-supporting processes. Around the world, the molecular methodologies are undergoing constant improvement and advancement to better serve the assessment and surveillance of microbial water quality.

Next generation sequencing (NGS) technology has been increasingly applied in assessing aquatic microbial community changes in response to different environmental stresses/pollutants and can provide a detailed understanding of how the various microbial ecosystems adapt to them. Sun et al. investigated the bacterial structure of different ecological niches in polluted urban lakes using 16S rRNA high-throughput sequencing. The study revealed distinct interaction patterns of bacterial communities in different niches and identified the important role of ecological niches in shaping bacterial responses to water pollution. By applying 16S and 18S rRNA gene amplicon sequencing, Wu et al. characterized the assembly of bacteria and protists in karst river and explored the adaptability of the abundant and rare bacterial and protistan subcommunities to environmental disturbances in studied water. They discovered that the abundant bacterial subcommunities exhibited a superior environmental adaption potential than the other subcommunities examined. However, the rare subcommunity played important role in maintaining the stability of the microbial communities.

Lately, with the advent of revolutionized CRISPR technology, novel molecular diagnostic tool based on CRISPR platform, e.g., SHERLOCK (Specific High Sensitive Enzymatic Reporter UnLOCKing) has been emerged and utilized for diverse environmental quality surveillance enabling rapid and accurate detection of the different targets. Wang et al. developed a Cas12a-based method to detect *Karenia mikimotoi*, a dinoflagellate responsible for harmful algal blooms. By recording the fluorescence signal released upon the successful cleavage of the target molecular, the optimized approach managed to deliver an easy and sensitive detection of *K. mikimotoi* within an hour. Similarly, by implementing CRISPR/Cas13a system in combination with RPA amplification, Li et al. demonstrated a well-functioned method for detection of grass carp reovirus (GCRV), causing serious hemorrhagic disease in grass carps. The established rapid method exhibited great specificity and sensitivity which was comparable with qPCR assays with over 90% congruency. Due to the streamlined system, the method holds great potential for field-deployment.

Additionally, recently the integrated assessment tool/modeling has gained growing attention for evaluating and predicting microbial water quality and health risks. Gitter et al. developed a novel qPCR-based microbial source tracking (MST) (targeting human, dog and gull) combined with a quantitative microbial risk assessment (QMRA) approach to estimate the potential pathogen risk of surveyed recreational beaches. The employed QMRA simulations revealed that human fecal source was the major driver of the high gastrointestinal illness risk. The study highlighted the importance of using QMRA and MST together for effective and informed beach management. Such combined approach has also been employed by Rytkönen et al. in their surface and bathing waters quality survey in Finland. Based on MST analyses and scenario-based QMRA assessment, they found that wastewater contamination increased the risk of gastroenteritis, such as norovirus GII infection, while animal farm contamination contributed largely to *Campylobacter jejuni* infection risk. Meanwhile, some wastewater treatment measures, such as wetland and UV-LED disinfection were found to be able to reduce the gastroenteritis risks effectively. Based on their research outcomes, it is recommended to implement QMRA and MST approaches for health risk evaluations of bathing water particularly under remarkable impacts of wastewater discharge and water runoffs (e.g., animal farms).

In conclusion, molecular diagnostics tools are essentially important for rapidly and reliably assessing the microbial water quality in a broad perspective, e.g., censoring aquatic ecosystem functioning under varying pollution pressures and raised health risks to humans and animals. With constant efforts devoted to the methodology development, molecular tools have been under continuous improvement and advancement. (Beyond) state-of-the-art techniques and evaluation systems have been increasingly adopted and enhanced the detection robustness, e.g., NGS-powered metagenomics, CRISPR-based pathogen detecting platform, QMRA and QMST risk assessment tool, etc. Apparently, there is no single method that can universally solve all related issues within this theme. Thus, different methods should be employed and applied coordinately to achieve effective and robust diagnostic purpose. Furthermore, the implementation of these advanced techniques must be accompanied by rigorous verification and validation tests to ensure their effectiveness and reliability in diverse environmental contexts with different local conditions.

## Author contributions

LP: Writing – original draft, Writing – review & editing. CG-A: Writing – original draft, Writing – review & editing.

